# Hybrid nanocomposite conduit based on oxidized polyvinyl alcohol and multiwalled carbon nanotubes: a new device supporting peripheral nerve regeneration in animal model of disease

**DOI:** 10.1093/rb/rbaf108

**Published:** 2025-11-12

**Authors:** Elena Stocco, Silvia Barbon, Marta Confalonieri, Lucia Petrelli, Alice D’Osualdo, Ludovica Ceroni, Stefania Benazzato, Martina Contran, Aron Emmi, Cesare Tiengo, Raffaele De Caro, Veronica Macchi, Silvia Todros, Piero G Pavan, Enzo Menna, Andrea Porzionato

**Affiliations:** Section of Human Anatomy, Department of Neuroscience, University of Padova, Padova, 35121, Italy; Department of Women’s and Children’s Health, University of Padova, Padova, 35128, Italy; Department of Surgery, Oncology and Gastroenterology, University of Padova, Padova, 35122, Italy; Foundation for Biology and Regenerative Medicine, Tissue Engineering and Signaling-TES, Onlus, Padova, 35121, Italy; Section of Human Anatomy, Department of Neuroscience, University of Padova, Padova, 35121, Italy; Foundation for Biology and Regenerative Medicine, Tissue Engineering and Signaling-TES, Onlus, Padova, 35121, Italy; Section of Human Anatomy, Department of Neuroscience, University of Padova, Padova, 35121, Italy; Department of Industrial Engineering, University of Padova, Padova, 35131, Italy; Section of Human Anatomy, Department of Neuroscience, University of Padova, Padova, 35121, Italy; Section of Human Anatomy, Department of Neuroscience, University of Padova, Padova, 35121, Italy; Department of Chemical Sciences, University of Padova & INSTM, Padova, 35131, Italy; Department of Chemical Sciences, University of Padova & INSTM, Padova, 35131, Italy; Section of Human Anatomy, Department of Neuroscience, University of Padova, Padova, 35121, Italy; Section of Human Anatomy, Department of Neuroscience, University of Padova, Padova, 35121, Italy; Plastic and Reconstructive Surgery Unit, Department of Neuroscience, University of Padova, Padova, 35121, Italy; Section of Human Anatomy, Department of Neuroscience, University of Padova, Padova, 35121, Italy; Section of Human Anatomy, Department of Neuroscience, University of Padova, Padova, 35121, Italy; Department of Industrial Engineering, University of Padova, Padova, 35131, Italy; Department of Industrial Engineering, University of Padova, Padova, 35131, Italy; Department of Chemical Sciences, University of Padova & INSTM, Padova, 35131, Italy; Section of Human Anatomy, Department of Neuroscience, University of Padova, Padova, 35121, Italy

**Keywords:** oxidized polyvinyl alcohol, carbon nanotubes, nerve conduits, peripheral nerve injuries, nerve regeneration

## Abstract

Severe peripheral nerve injuries represent a significant clinical problem, and intense efforts are dedicated toward the identification of the ‘ideal’ nerve conduit (NC). In this context, incorporating electrical cues within the device wall seems to be extremely appealing. Here, a new NC based on the new polymer oxidized polyvinyl alcohol (OxPVA) (oxidation degree 1%) + water-soluble multiwalled carbon nanotubes (MWCNT-S) (0.1 wt% in OxPVA) was developed and characterized for ultrastructure and mechanical behavior. Subsequently, OxPVA+MWCNT-S NCs were implanted in animal model of disease (Sprague–Dawley rat; sciatic nerve, gap: 5 mm) and compared with OxPVA and Reverse Autograft. Following sciatic functional index evaluation, implants-associated outcomes were verified on explants through histology, immunohistochemistry, immunofluorescence and morphometric studies on semithin sections, after 6 weeks from surgery. According to preclinical study evidence, all the NCs supported nerve regeneration (S100/β-tubulin/neurofilaments) without severe inflammatory reaction (CD3/F4/80). Morphometric studies showed the highest cross-section area and fascicular area for Reverse Autograft followed by OxPVA+MWCNT-S and OxPVA. The epineurium thickness was the highest in Reverse Autograft followed by OxPVA and OxPVA+MWCNT-S. Myelinated axon density was highest for OxPVA+MWCNT-S, followed by OxPVA and Reverse Autograft; myelinated axons total number followed this descending order Reverse Autograft˃OxPVA+MWCNT-S˃OxPVA. Additionally, the g-ratio distribution highlighted a similar trend for OxPVA+MWCNT-S and Reverse Autograft with most nerve fibers within the 0.6–0.7 interval. Atrophy of the operated-limb gastrocnemius was comparable in the whole cohort. Interestingly, MWCNT-S incorporation in OxPVA showed to be an appealing strategy to improve the morpho-structural outcomes associated with these devices.

## Graphical abstract

**Figure:**
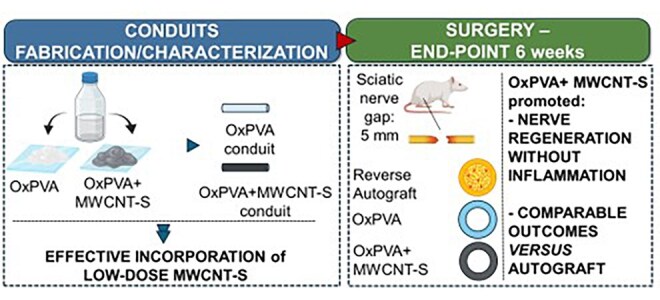

## Introduction

In the field of peripheral nerve injury repair, intense bioengineering research is devoted toward identification of devices able to guarantee for successful outcomes in case of neurotmesis, the most critical surgical scenario associated with a complete interruption of the nerve continuity, complete loss of function and limited axonal regrowth. Currently, when direct end-to-end repair is not feasible, autograft placement remains the gold-standard treatment option. However, the risk of donor-site morbidity and dimensional mismatch between donor and recipient nerves have further driven research into off-the-shelf alternatives. Among the eleven Food and Drug Administration-approved NCs [1, 2], none stands out for the *in vivo* outcomes. Synthetic bioresorbable NCs may lead to an immune response, scar tissue formation and release of by-products that are detrimental for the regeneration process. Non-biodegradable NCs require a second surgery for their removal, with further disadvantage [3]. Within this scenario, many materials, differently improved, have been investigated to ameliorate NCs performances. The main challenge consists in creating conduits with the appropriate chemical, physical and structural properties to withstand the mechanical stresses experienced in the body and to remain functional during critical stages of nerve healing and recovery [4].

Experimental evidence highlights that promoting the electrical conductivity of devices for peripheral nerve regeneration enhances the potential for nerve regrowth and remyelination with complete lesion recovery. This is consequence of improved electrostatic cell–cell and cell–scaffold interactions [5, 6]. Among the conductive materials used for artificial NCs fabrication are encountered: polypyrrole (PPy), polyaniline (PAni) and polythiophene (PTh); however, their rigid structure obstacles their performance [7]. Along with conductive polymers, carbon nanotubes (CNTs), in either a single-wall (SWCNT) or multi-wall (MWCNT) configuration, have a wide range of applications in bioengineering, including the development of NCs. In this context, they have demonstrated the ability to support sustainable neuronal survival and promote neuronal outgrowth [8–12]. Specifically, they are recognized as interesting nanoscale candidates for incorporation into polymeric networks, thus leading to nanocomposite materials [13]. However, because of their poor dispersion in aqueous medium or other solvents, tailored chemical modifications of CNT structures are necessary to prevent aggregation and potential toxicity issues, while preserving their ability to promote neuronal differentiation.

Among polymers with appealing characteristics for tissue engineering purposes,our partially oxidized polyvinyl alcohol (OxPVA) revealed as a captivating biomaterial [14–16] also for the development of biodegradable nerve conduits/wraps [17–21] eventually functionalized by growth factors adsorption (nerve growth factor (NGF); Ciliary Neurotrophic Factor (CNTF) with/without the transactivator transduction domain (TAT)) or by self-assembling peptides mechanical incorporation (EAK; EAK-YIGSR) [2, 17, 20, 22]. Additionally, new OxPVA composites with water-soluble MWCNTs (OxPVA+MWCNT-S) were also fabricated and characterized [23–25]; however, no evidence was provided about their ability in promoting nerve regeneration *in vivo*.

In the present experimental work, it was investigated the effect of OxPVA+MWCNT-S NCs in nerve regeneration after neurotmesis (animal model: Sprague–Dawley rats, sciatic nerve injury, gap: 5 mm). OxPVA conduits free from MWCNT-S and Reverse Autograft were used as control groups. Study evidence confirmed that mechanical incorporation of cues within OxPVA is a simple and effective method for polymer customization. Moreover, OxPVA activation by MWCNT-S showed to improve the hydrogel performances with appealing outcomes *in vivo.*

## Materials and methods

### Preparation of OxPVA

The OxPVA was prepared according to a well-established protocol, as described by Stocco *et al.* [15]. A weighed amount of PVA powder [molecular weight (Mw) 146 000–186 000 Da, 99+% hydrolyzed] was suspended in MilliQ water and solubilized. Hence, partial oxidation was performed by adding an acidic potassium permanganate (KMnO_4_) solution. Oxidized polymer solution underwent extensive dialysis (8000 Da cut-off (Sigma-Aldrich)); then, it was frozen at −20°C overnight and lyophilized (Speed Vac Concentrator Savant, Instruments Inc., Farmingdale, NJ, USA) for long-term storage. For polymer recovery, 16 weight (wt) % OxPVA was weighed, suspended into MilliQ water and heated in boiling bath.

### MWCNT functionalization

MWCNT (ACS Material LLC, OD: <8 nm, SKU# CMP00105) were functionalized according to a previously reported procedure [25] here summarized. MWCNT (50 mg, 4.16 mmol of C) were dispersed in an aqueous solution of sodium sulfanilate dihydrate (48.1 mg, 0.05 eq/mol C) (99%, Sigma-Aldrich). Then, isopentyl nitrite (58 µL, 0.1 eq/molC) (96%, Sigma-Aldrich) was added and the reaction proceeded for 4 h at 80°C under N_2_ flux. The product was recovered by filtration and washed with water and methanol.

### Nerve conduits fabrication

Three grams of the OxPVA hydrogel were combined with MWCNT-S suspension (0.1 wt% in OxPVA). The homogeneous distribution of the MWCNTs within the polymer matrix was achieved by mechanical embedding. Subsequently, the NCs were fabricated by cast-moulding method, aspiring the OxPVA+MWCNT-S into a tubular stainless mold (length: 5 cm; internal diameter: 2.1 mm) equipped with a central coaxial mandrel (diameter: 1.2 mm). Thereafter, the system underwent to six freeze-thawing (FT) cycles (one cycle: freezing (at −20°C for 6 h)/thawing (at 22°C for 1 h)) leading to polymer physical cross-linking and NCs set-up. The NCs were stored at −20°C until use.

The same procedure was carried out to obtain OxPVA scaffolds (MWCNT-S-free) used as controls.

### Nerve conduits characterization

#### Macroscopic appearance and ultrastructure

OxPVA and OxPVA+MWCNT-S NCs were compared for their gross appearance to detect differences ascribable to MWCNT-S presence within the OxPVA matrix. The samples were then processed for scanning electron microscopy (SEM) analysis to characterize their ultrastructure. Briefly, nerve conduits were cut in ∼ 0.5 mm long segments and fixed in 2.5% glutaraldehyde solution in 0.2 M phosphate buffer saline (PBS) (pH 7.4, 24 h). Thus, the samples were dehydrated with a graded ethanol series, exposed to critical-point drying and gold sputtering and finally observed by a SEM system JSM-6490 (Jeol USA, Peabody, MA, USA).

The spatial distribution of MWCNT-S inside the OxPVA was evaluated by transmission electron microscopy (TEM) according to the following procedure: the OxPVA+ MWCNT-S samples were fixed with 2.5% glutaraldehyde (EMS; Cat. No. 16220) in 0.1M sodium cacodylate buffer pH 7.4 ON at 4°C. Subsequently, the samples were post-fixed with 1% osmium tetroxide in 0.1M sodium cacodylate buffer for 1 hour at 4°C. After washing three times with water, samples were dehydrated in a graded ethanol series and embedded in an epoxy resin (Sigma-Aldrich 46345). Ultrathin sections (60–70 nm) were obtained with a Leica Ultracut EM UC7 ultramicrotome, counterstained with uranyl acetate and lead citrate and viewed with a Tecnai G2 (FEI) transmission electron microscope operating at 100 kV. Images were captured with a Veleta (Olympus Soft Imaging System) digital camera.

#### Suture retention tests

**Figure 1.**
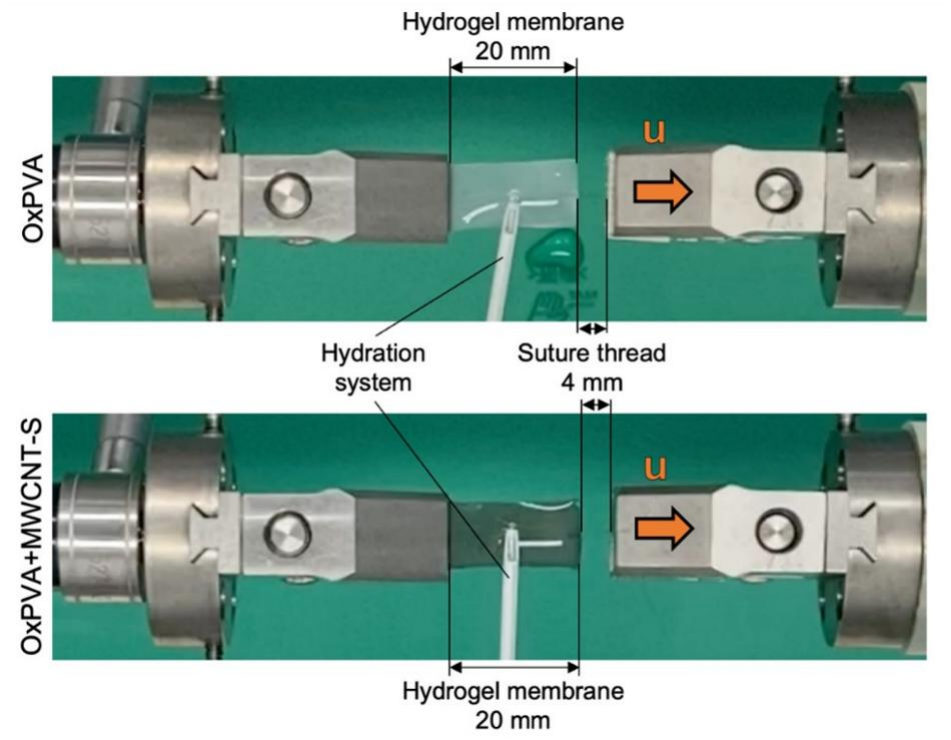
Suture retention test set-up. The initial gauge length is 24 mm (20 mm hydrogel membrane, 4 mm suture thread). Hydration of the sample was ensured by means of a drop-by-drop release of PBS solution. A displacement (u) of 24 mm is imposed at 0.12 mm s^−1^.

The resistance to suture pull-out of OxPVA and OxPVA+MWCNT-S hydrogels was tested with suture retention (SR) test (five samples/experimental group) using the Bose ElectroForce^®^ Planar Biaxial Test Bench instrument (TA Instruments, New Castle, USA) in uniaxial tensile mode. After cross-linking, the OxPVA and OxPVA+MWCNT-S hydrogels membranes were cut into rectangular samples (34×8×0.6 mm). The sample thickness was selected to be the same as the nerve conduit wall thickness, while the sample geometry was simplified for these tests only. The specimens were then sutured using Nylon 9–0 sutures; the thread was fixed to the grip and the opposite sample edge was clamped. The initial gauge length was equal to 24 mm, including 20 mm of hydrogel membrane and 4 mm of suture thread (Figure 1). All SR tests were performed in quasi-static condition, with elongation rate of 0.12 mm s^−1^ up to suture pull-out, in hydrated conditions. Suture Retention Strength (SRS) was determined from each test as the maximum force value reached during the suture pull-out.

#### Nerve conduits implantation in animal model of peripheral nerve injury

All animal procedures were approved by the ethical committee of Padua University, in agreement with the Italian Department of Health guidelines (Authorization n. 837/2019-PR, 9 December, 2019).

Fifteen adult Sprague–Dawley rats were randomly divided into three experimental groups (*n* = 5/each): Reverse Autograft, OxPVA and OxPVA+MWCNT-S. Briefly, the animals were anaesthetized with gas mixture of isoflurane/oxygen; thus, the left sciatic nerve was exposed and medially transected, leaving a gap of 5 mm which was surgically treated with the positioning of Reverse Autograft or conduits. Regarding the Reverse Autograft group, the flipped sciatic nerve segment (5 mm in length) was reimplanted using Nylon 9–0 sutures; as for the conduits (10 mm in length), they were coaxially interposed between the proximal and the distal stumps and sutured to the epineurium with Nylon 9–0 sutures. The incision was closed with silk sutures (4-0). After surgery, the animals were allowed to recover in the cage; over the next five days, they were given appropriate anti-inflammatory and antibiotic therapy (Rimadyl^®^ and Baytril^®^, respectively). The operated rats were housed in an adequate environment with a temperature-controlled facility, fed with laboratory rodent diet and given water *ad libitum*. Euthanasia was performed using carbon dioxide asphyxiation at the experimental endpoint of 6 weeks post-surgery. Initially, the implants were evaluated *in situ* for any potential ruptures and assessed for integrity, as well as the presence of any inflammatory signs. Subsequently, the implants were isolated/dissected and differently processed for subsequent analyses. Two samples/group were properly fixed for histological/immunohistochemical analyses/immunofluorescence study; whereas, three samples/group were processed for semithin sections preparation, morphometric study and TEM analysis. Additionally, the gastrocnemius muscles of the operated limbs were also considered for comparison, focusing on weight analysis.

### Animals’ wellbeing and functional recovery

#### Animals’ weight

To assess animals’ wellbeing, their weight was recorded at Day 0, Day 20 and Day 42 from surgery to highlight eventual weight loss amenable to pain symptoms associated with surgery.

#### Sciatic functional index assessment

The attribution of the sciatic functional index (SFI) score was adopted to evaluate the motor recovery of the operated sciatic nerve at six weeks from surgery. As previously described, the rats were placed in a hallway previously lined with white paper; thus, the animals were allowed to walk along it once their hind legs had been stained with black ink. The SFI was determined according to Bain *et al.* [26] formula considering the footprints of both the normal and operated paws. Score around 0 is associated with normal nerve function; score around −100 is associated with total motor sciatic nerve dysfunction.

#### Nerve conduits retrieval and surgical site analysis

At the experimental endpoint (6 weeks from surgery) euthanasia was administered. The implants were first considered *in situ* for presence of inflammation-related signs eventually associated with perineural adhesions. Thereafter, adherences were scored according to Petersen *et al.* [27]. Briefly, grade 1: ‘Minimal adhesion that did not require dissection or that required minimal dissection’; grade 2: ‘Moderate adhesions that required some vigorous blunt dissection but without sharp tools’; grade 3: ‘Significant adhesions that required sharp dissection’.

#### Histological characterization of the regenerated tissue

Once harvested, *n* = 2 samples/group were fixed in 10% (wt/v) formalin in PBS; hence, the central portion of the specimens was isolated, paraffin-embedded and sectioned (4-μm-thick serial transversal sections). Overall appearance and presence of fibro-connective tissue were analyzed by hematoxylin and eosin (H&E) and Azan Mallory, respectively.

### Immunohistochemistry

The central portions of the samples in cross-section were immunostained using the following antibodies: anti-CD3 (polyclonal rabbit anti-human CD3, A 0452; Dako^®^, Milan, Italy, diluted 1:500) and anti-F4/80 (polyclonal rabbit anti-mouse, sc-26643-R; Santa Cruz Biotechnology, CA, USA, diluted 1:800) to highlight lympho-macrophagic infiltration; anti-S100 (polyclonal rabbit anti-S100, Z 0311; Dako^®^, diluted 1:5000) to identify Schwann cells; anti-β-tubulin (polyclonal rabbit neuronal class III β-tubulin, PRB-435P; Covance, Princeton, NJ, USA, diluted 1:1000) and anti-neurofilament heavy chain (NEFH) antibodies (rabbit recombinant monoclonal neurofilament heavy polypeptide antibody, ab207176; Abcam, Cambridge, UK, diluted 1:1000) to confirm the presence of axons. Except for S-100, antigen unmasking was done (10 mM sodium citrate buffer, pH 6.0, at 90°C for 10 min): sections were first incubated in blocking serum [0.04% (wt/v) bovine serum albumin (BSA; A2153, Sigma-Aldrich, Milan, Italy) and 0.5% (wt/v) normal goat serum (X0907, Dako^®^)] to assure unspecific binding removal (30 min at room temperature, RT) and then with the above primary antibodies (1 h at RT). Primary antibody binding was revealed by incubation with anti-rabbit/mouse serum diluted 1:100 in blocking serum (30 min at RT) (Dako^®^ EnVision + TM peroxidase, rabbit/mouse; Dako^®^, Glostrup, Denmark) and developed in 3,3-diaminobenzidine (3 min at RT). Finally, hematoxylin was adopted for counterstaining. As a negative control, incubation without primary antibodies was performed.

### Immunofluorescence

The quality of the regenerated tissue was verified on longitudinal nerve sections through immunofluorescence (IF). To this purpose, S100 (polyclonal rabbit anti-S100; Z 0311; Dako^®^, diluted 1:3000), and PGP9.5 (polyclonal mouse anti-PGP9.5; Dako^®^, diluted 1:300) markers were used. Once unmasked (10 mM sodium citrate buffer, pH 6.0, at 95°C for 10 min), the samples were dipped in quenching solution (133.76 mg of NH_4_Cl in 50 ml dH_2_O for 10 min at RT) to reduce native molecules’ background fluorescence. Therefore, after PBS washing, samples were first treated with permeabilization and blocking solution (15% vol/vol Goat Serum, 2% wt/vol BSA, 0.25% wt/vol gelatin, 0.2% wt/vol glycine in PBS) containing 0.5% Triton X-100 (1 h at RT) and then incubated with the primary antibodies (overnight at 4°C). Alexa-Fluor plus 568 anti-Rabbit secondary antibody (A11011) (red) and Alexa-Fluor plus 488 Goat anti-Mouse secondary antibody (A32723) (green) were diluted 1:200 in 5%BSA + 0.05% Triton-X solution (30 min at RT) and incubated for 1 h at RT. Hoechst (Invitrogen, dilution: 1:10 000 in PBS) was used to stain the nuclei in blue. The positive signals were detected and acquired with a Leica, SP5 Laser Scanning Confocal Microscope.

### Morphometric study

The regenerative outcome associated with the different implants was verified through a morphometric study. In accordance with a previously reported method [18], *n* = 3 samples/group were preliminarily fixed in 2.5% (wt/v) glutaraldehyde in 0.1 M PBS, divided in four different segments and post-fixed in 1% osmium tetroxide (Agar Scientific Elektron Technology, UK) in 0.1 M phosphate buffer. After dehydration in a graded alcohol series, the samples underwent epoxy resin embedding; semi-thin sections (0.5 μm) were cut through an ultramicrotome RMC-PTX PowerTome (Boeckeler Instruments, AZ, USA) and then stained with 1% toluidine blue. Images, acquired with a Leica DMR microscope (Leica Microsystems Wetzlar, Germany), were considered for the morphometric study. Specifically, central transversal sections were compared for: mean total cross-section area [µm^2^], fascicular area [µm^2^] and epineural sheath area (%) ([total cross-section area−fascicular area]×100). Contextually, five random quadrants from the fascicular area were selected and three high-power fields (7523 µm^2^/quadrant) were focused to calculate total myelinated axons number, average myelinated axons density (axons/µm^2^) and g-ratio (axon diameter/fiber diameter). ImageJ software was used for measurements.

Additionally, ultrathin sections (60 nm) were also collected on 300-mesh copper grids, counterstained with 2% (wt/v) uranyl acetate and then with Sato’s lead prior to be observed by a Hitachi H-300 Transmission Electron Microscope (TEM).

### Gastrocnemius muscle analysis

Both sides gastrocnemius muscles were carefully dissected, and their wet weights (g) were recorded with an electronic balance. Left gastrocnemius weight was expressed as a percentage of contralateral control.

### Statistical analysis

Results are presented as the mean±SD of at least three replicates for each experiment. Statistical analysis was conducted using Prism software (version 9.3.1, GraphPad Software, San Diego, USA). Differences between experimental groups were analyzed using both parametric and non-parametric statistical methods. One-way ANOVA followed by Tukey’s *post hoc* test was used for normally distributed data, while the Kruskal–Wallis test followed by Dunn’s *post hoc* test was applied for non-normally distributed data. Statistical significance was set at *P* ≤ 0.05.

SRS values of OxPVA and OxPVA+MWCNT-S hydrogels were analyzed through a non-parametric Mann–Withney *U* test, considering a significant *P*-values lower than 0.05. Statistical analyses were carried out by means of MATLAB (version: 9.14.0 (R2023a), Natick, Massachusetts).

## Results

### Distribution of MWCNT-S in OxPVA

**Figure 2.**
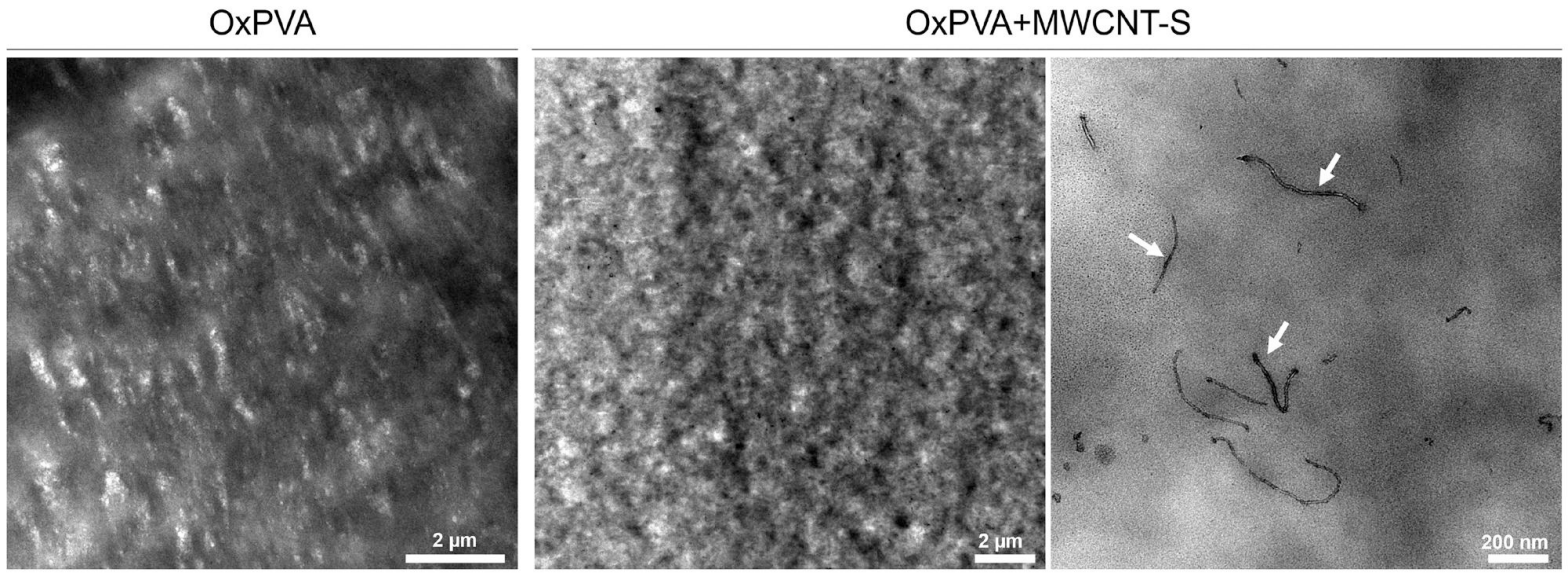
TEM images of OxPVA and OxPVA+ MWCNT-S samples acquired at different magnifications. White arrows show MWCNT-S within the polymer matrix. Scale bars: 2 μm; 200 nm.

TEM analysis was performed on OxPVA and OxPVA+ MWCNT-S samples to evaluate the spatial distribution of MWCNT-S within the polymer matrix. From the images, MWCNT-S appear rather uniformly distributed as individual tubes (Figure 2). This evidence provides further proof that our functionalization approach prevents CNT aggregation not only in solution, but also in the solid matrix, thus enhancing potential effects of the filler on the physical properties of the nanocomposite, as already shown in similar cases [28]. Indeed, when CNTs are present as individual tubes rather than aggregates, the interfacial area between filler and matrix is highly enhanced and a much lower loading is required to reach a conduction percolative network.

### Nerve conduits appearance

**Figure 3.**
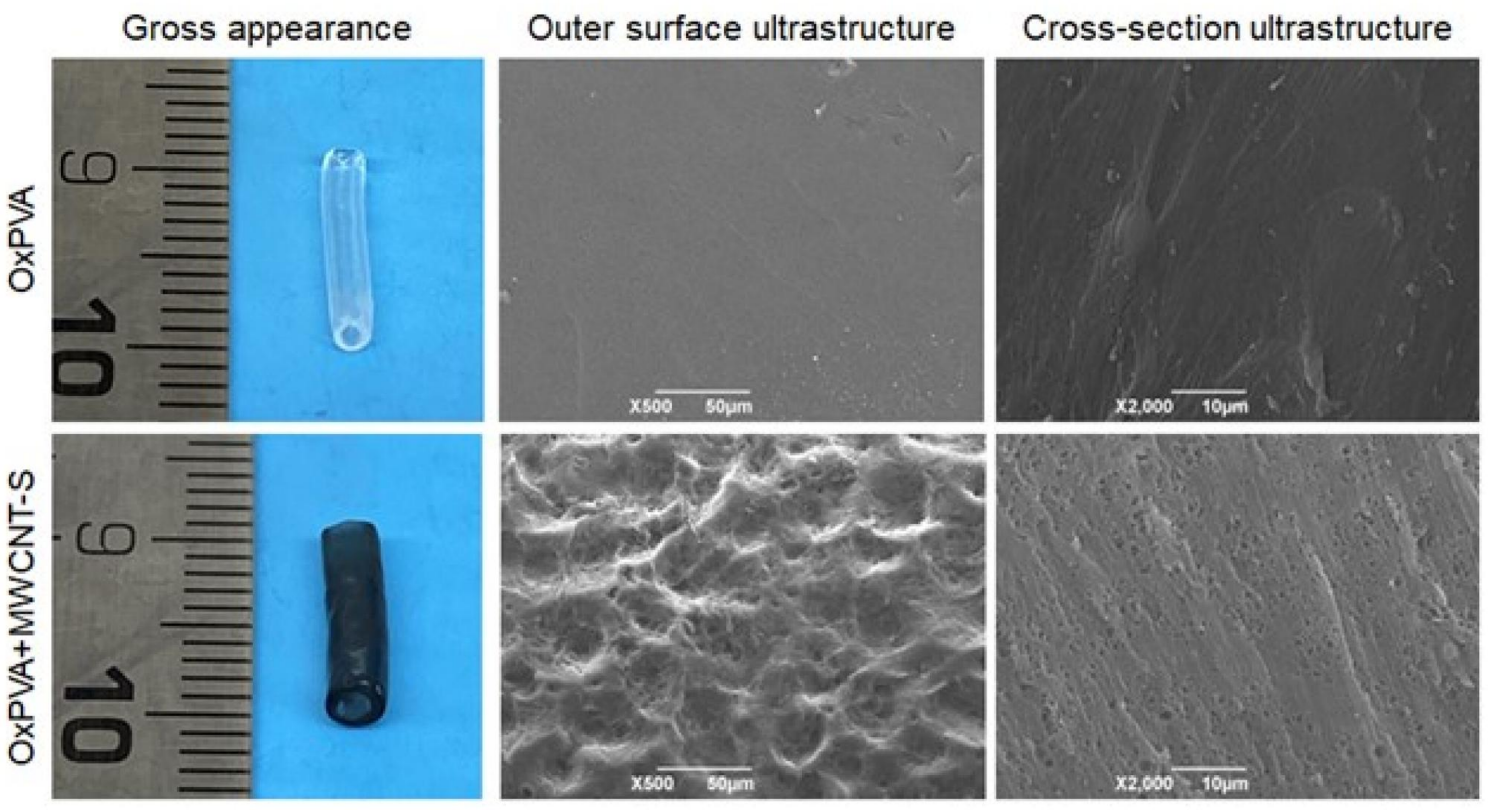
OxPVA and OxPVA+MWCNT-S nerve conduits gross appearance and ultrastructure referring to both the outer surface (scale bar: 50 μm) and the cross-section (scale bar: 100 μm).

The overall characteristics of the devices were initially assessed through comparison of their macroscopic appearance and ultrastructure. Following FT and once extruded from the mold, the devices appeared manipulable while maintaining their patency. The presence of MWCNT-S imparted a distinct dark color to the composite NCs; no CNT agglomeration areas, typically appearing as darker spots, were detected, suggesting nanostructures homogeneous dispersion within the polymer matrix. At SEM analysis, OxPVA conduits displayed a smooth outer surface and cross-section, whereas MWCNT-S imparted certain irregularities in the outer wall together with nanopores in section (Figure 3).

### Suture retention strength

**Figure 4.**
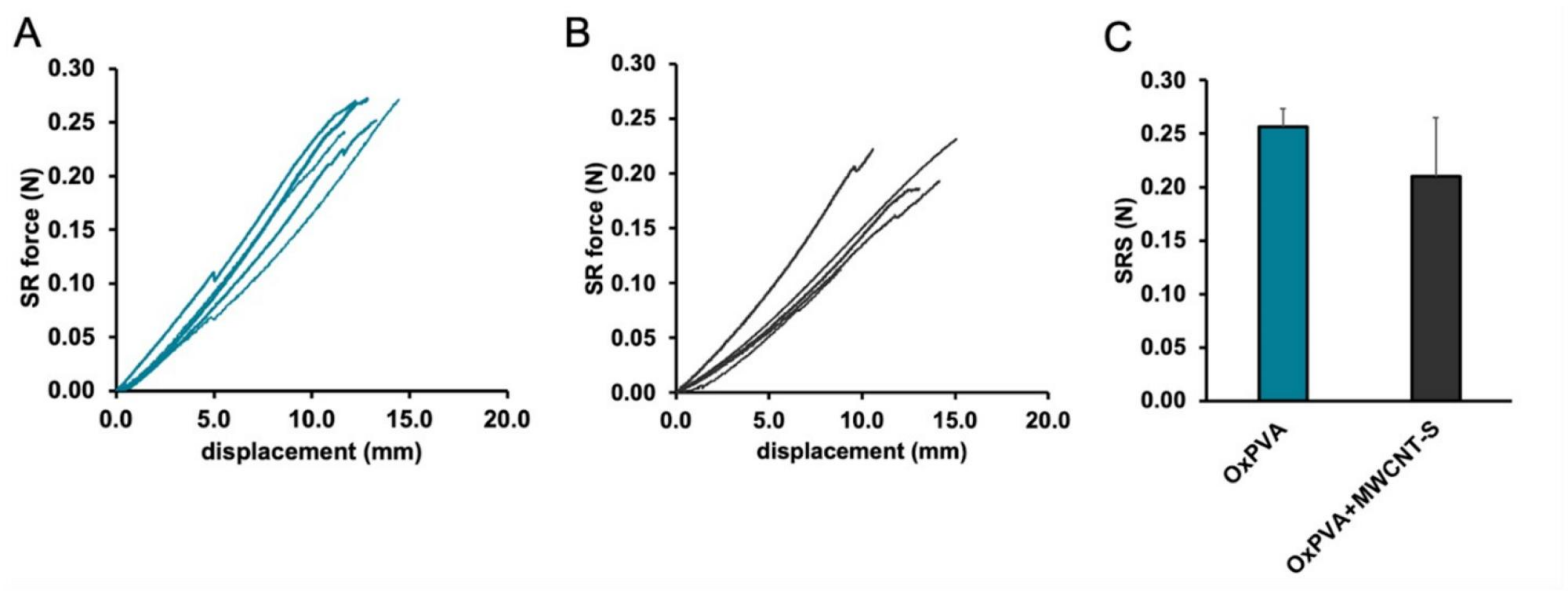
Suture retention force vs displacement of OxPVA (**A**) and OxPVA+MWCNT-S (**B**) up to suture pull-out. Comparison of suture retention strength values (±SD) for OxPVA and OxPVA+MWCNT-S (**C**).

The results of SRS on OxPVA and OxPVA+MWCNT-S membranes are shown in Figure 4. These tests allowed to identify a range of SRS between 0.23 and 0.27 N for OxPVA, and between 0.12 and 0.23 N for OxPVA+MWCNT-S. No statistical difference was detected between the SRS of the two experimental groups (*P*-values >0.05).

### Surgical repair of injured sciatic nerve

**Figure 5.**
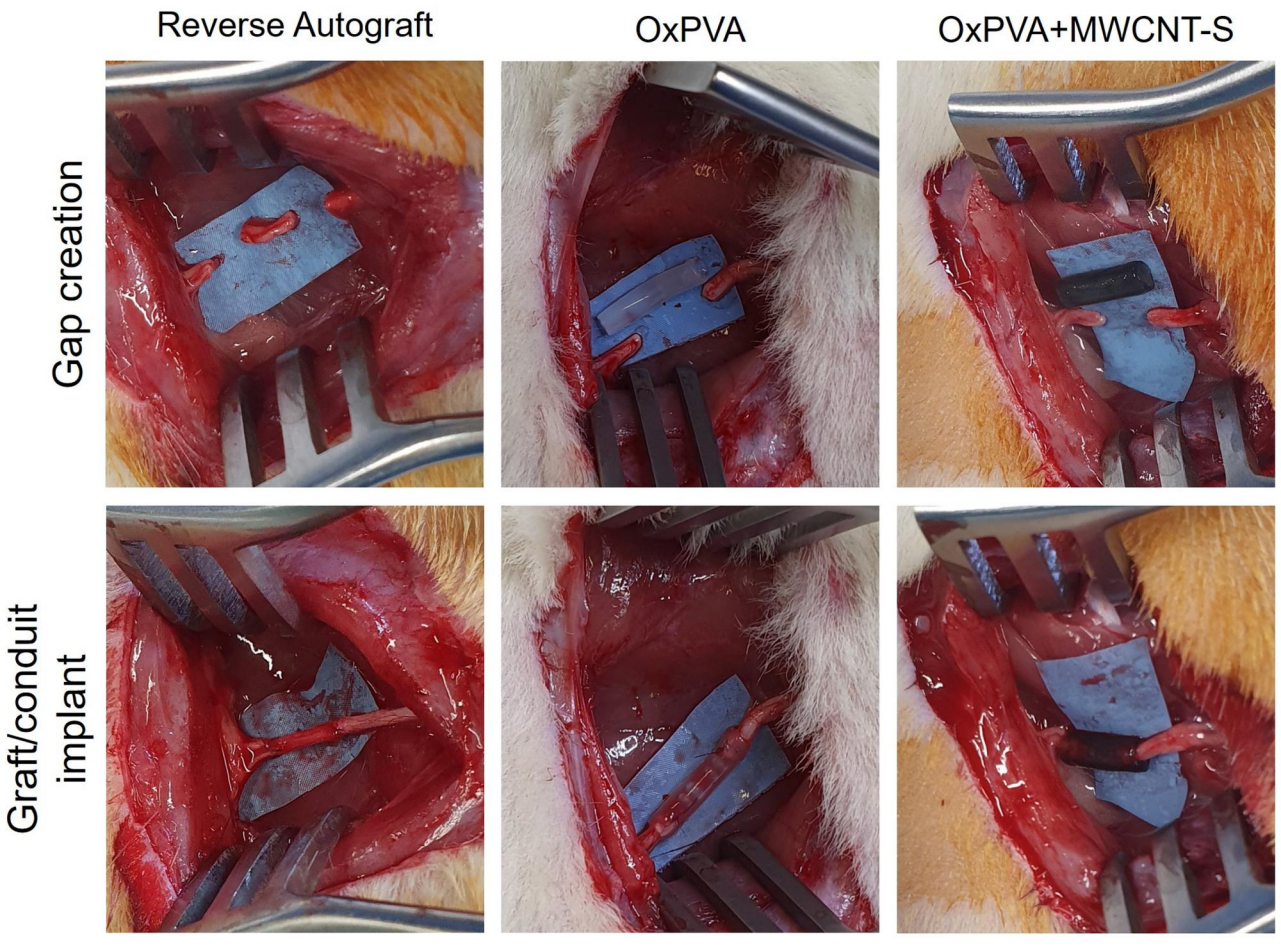
Reverse autograft, OxPVA and OxPVA+MWCNT-S nerve conduits implantation in Sprague–Dawley rat sciatic nerve lesion model (gap: 5 mm).

The OxPVA conduits demonstrated satisfactory performance once placed *in situ*, fitting well with the nerve ends. Additionally, no ruptures occurred during suturing, and no conduit kinking was observed. The same was verified for the composite devices, OxPVA+MWCNT-S. Certainly, the transparent appearance of OxPVA conduits favored their placement *in vivo*; the stumps correct positioning was perceivable for OxPVA+MWCNT-S devices (Figure 5).

### Behavioral response to implants and functional recovery assessment

**Figure 6.**
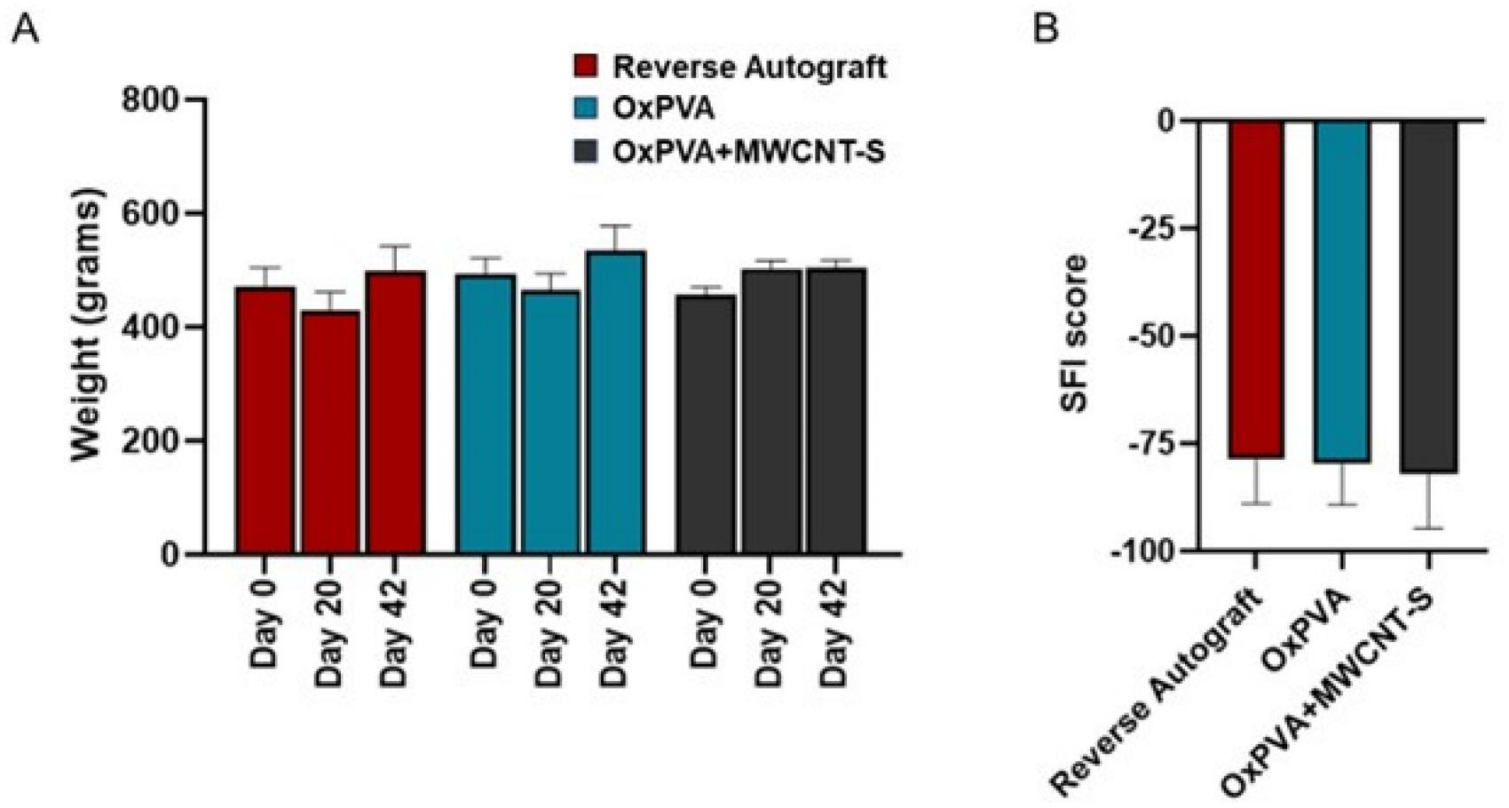
Animals’ weight and functional recovery assessment. Animals’ weight trend was evaluated at the day of surgery (Day 0), halfway through the experimental period (Day 20) and before the experimental endpoint (Day 42) (**A**). Sciatic functional index (SFI) score attribution before euthanasia. SFI around 0 suggests a normal nerve function; SFI around −100 is associated with total motor sciatic nerve dysfunction (**B**). No statistically significant differences were observed comparing the experimental groups in both weight and SFI score.

Animals’ wellbeing evaluation is an important index to determine surgery toleration and weight is a fundamental indicator of animals’ overall health. Weight monitoring from Day 0 up to Day 42 showed no significant variation within the cohort (Figure 6A).

Nerve function recovery was assessed by the SFI score attribution. At 6 weeks, the SFI measured values were −78.70 ± 10.30 for Reverse Autograft; −79.52 ± 9.73 for OxPVA and −82.20 ± 12.48 for OxPVA+MWCNT-S (Figure 6B).

### Evaluation of retrieved samples

Following euthanasia and sciatic nerve exposure, the implants were observed *in situ*. According to the adhesions’ score, adherences at the implants were graded as 2 for the whole cohort. Following the thin connective sheath removal, the conduits appeared as well integrated with the nerve, proving the excellent ability of the devices to be stable once positioned in a high mobile region like that of the sciatic nerve; furtherly, the overhangs of the conduits well supported the suture to the nerve stump’s epineurium without dislocation signs. Neither scar tissue nor neuroma were detected.

**Figure 7.**
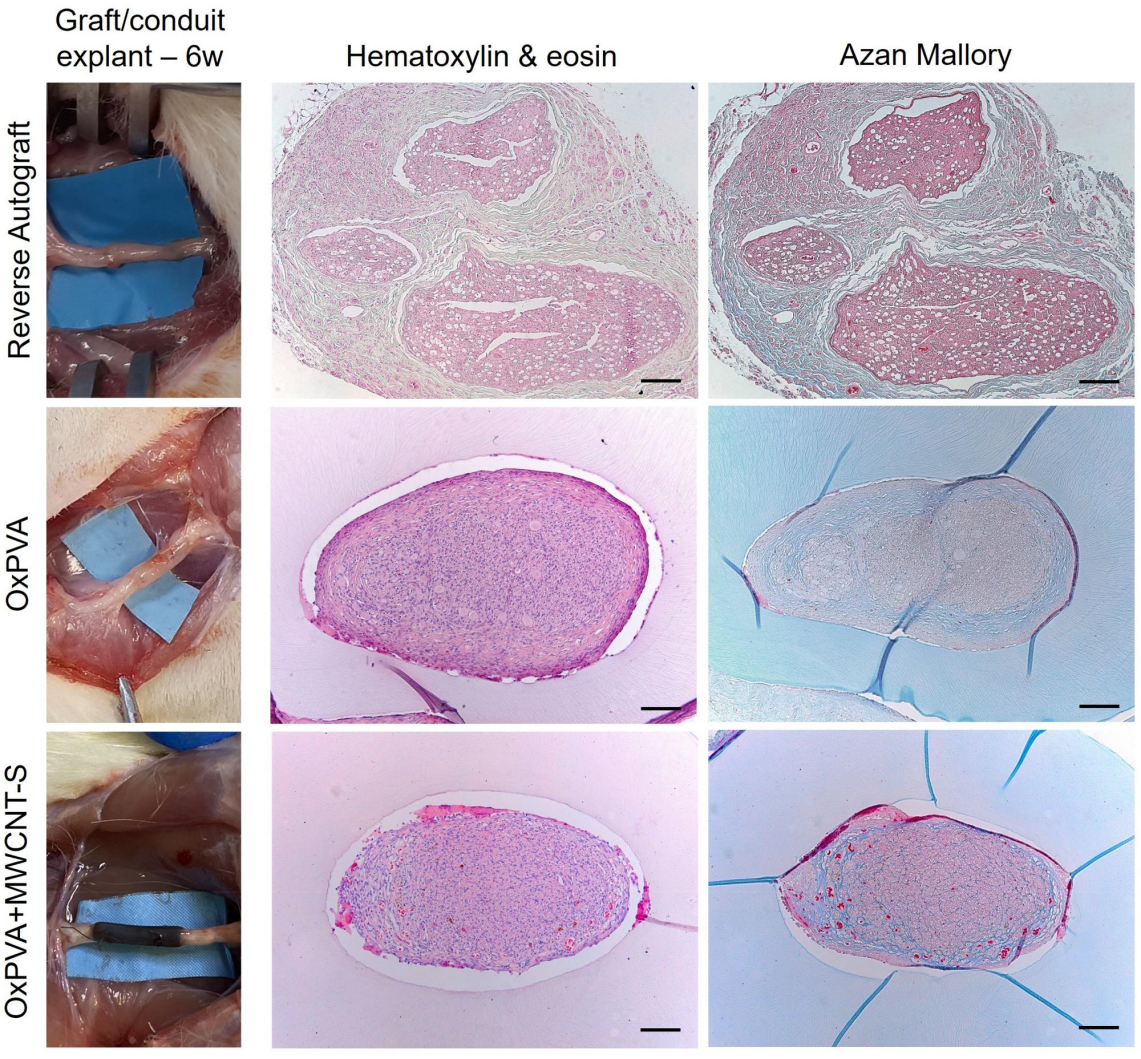
Reverse autograft and OxPVA-based nerve conduits at 6 weeks from surgery. Hematoxylin & eosin and Azan Mallory staining showing the overall appearance and fibro-connective tissue presence/distribution in correspondence of the Central sections of the explants. Scale bar: 100 μm.

Once excised, the NCs all showed a regenerated tissue inside that was macroscopically identifiable also through the black walls of the OxPVA+MWCNT-S conduits. Explants examination by H&E confirmed this evidence; in parallel, Azan Mallory staining highlighted the presence of connective tissue (i.e. collagen fibers, blue-stained) forming both the epineurium and an initial septal organization inside the newly regenerated tissue, likely suggesting the tissue organization in fascicles. Histological analysis showed Reverse Autograft overall appearance, exhibiting three fasci separated by fibro-connective tissue (Figure 7).

**Figure 8.**
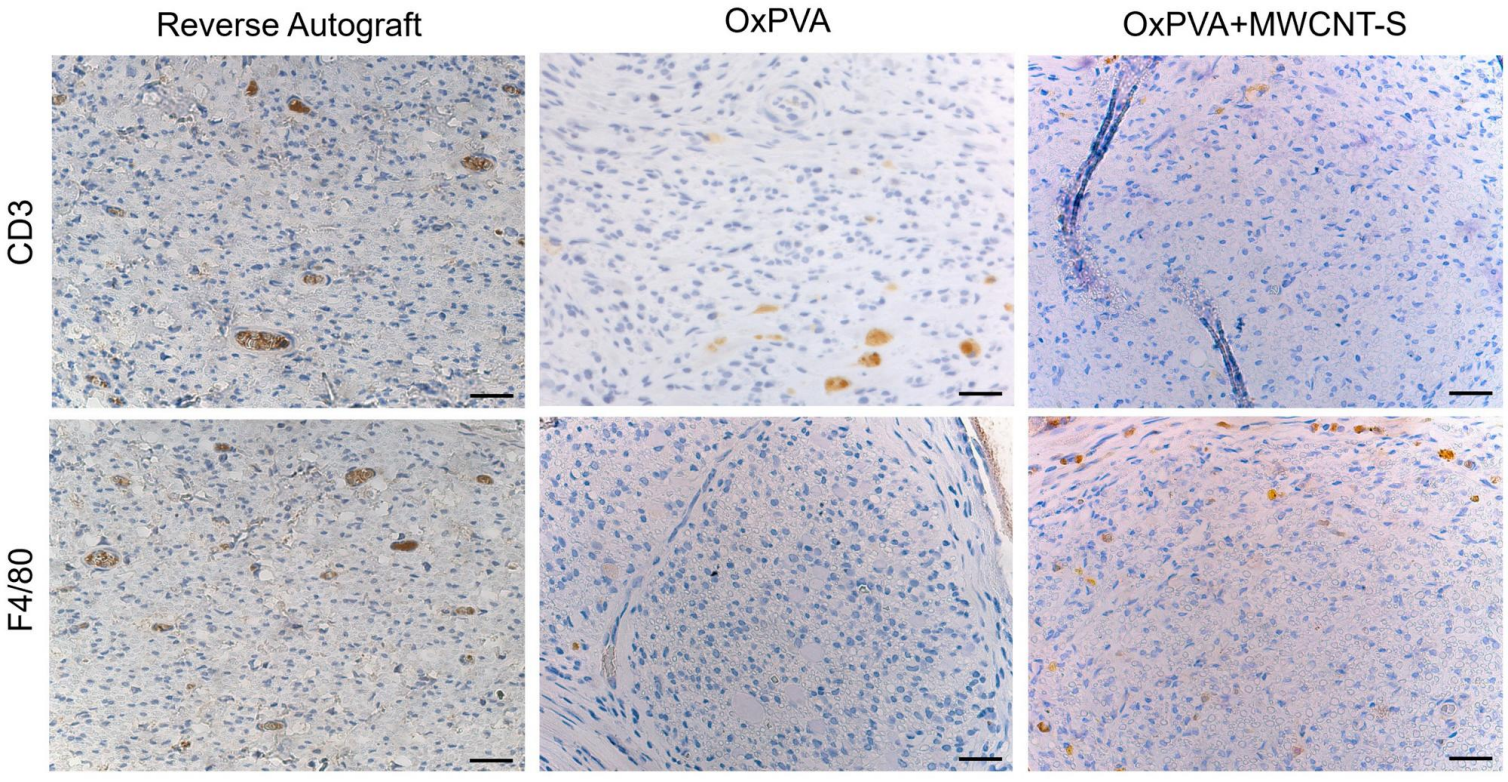
Immunolocalization of CD3 and F4/80 positive elements at the Central portion of the explants to highlight eventual presence of lymphocytes and macrophages, respectively. Scale bar: 25 μm.

In accordance with immunohistochemistry-based evidence, only few CD3 and F4/80 immunopositive elements were detected inside NCs. Regarding the Reverse Autograft group, lymphocytes and macrophages were scant and mainly identifiable in correspondence of vessels (Figure 8).

**Figure 9.**
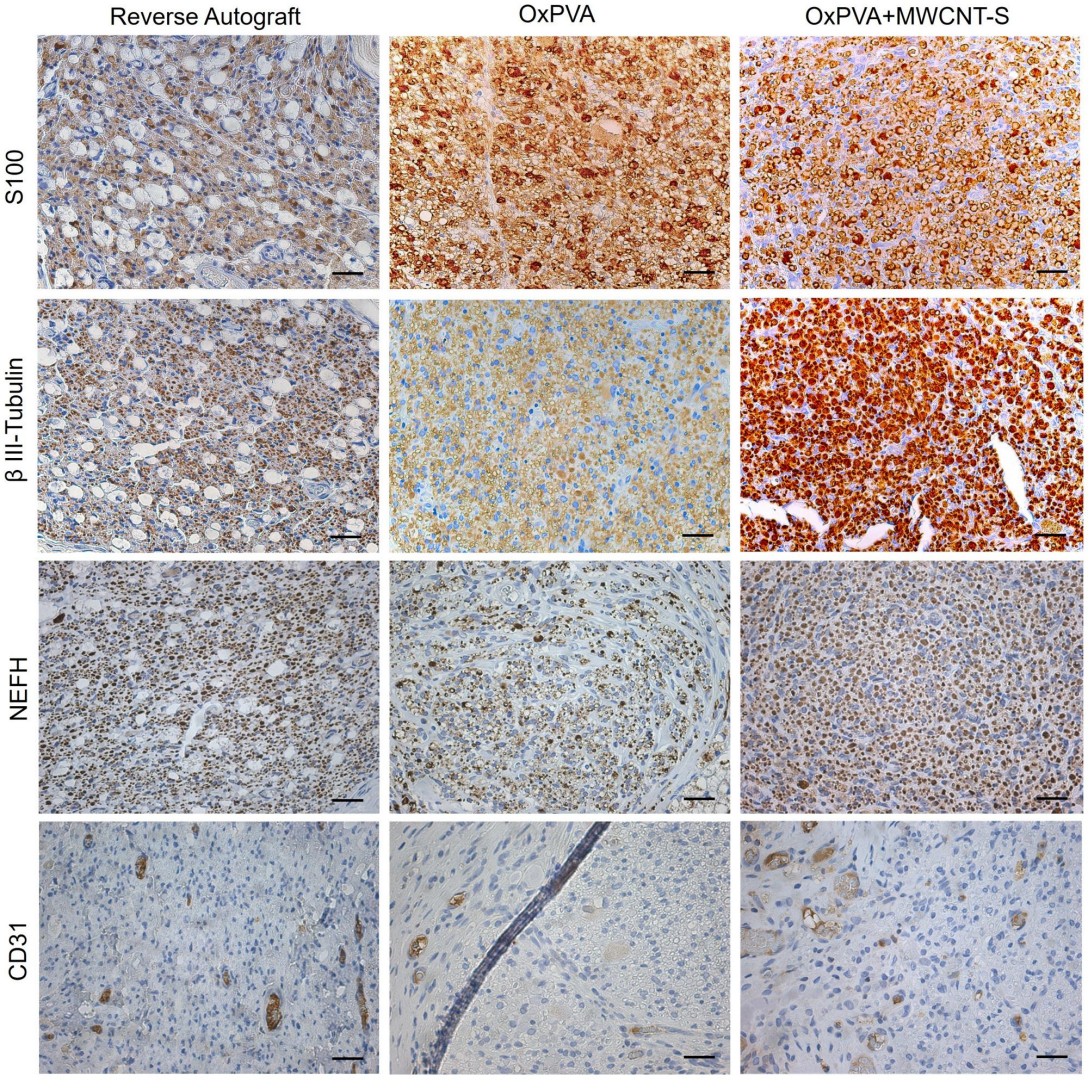
Immunolocalization of S-100 (Schwann cells), β III-tubulin (axons), NEFH (neurofilament heavy chain) and CD31 (endothelial cells) at the Central portion of the explants. Scale bar: 100 μm.

Central portions all showed a manifested immunoreactivity for the Schwann cells marker S100 and for the axonal markers β III-tubulin and NEFH. Additionally, immunostaining for the endothelial cell marker protein CD31 allowed for detection of some positive elements too (Figure 9).

**Figure 10.**
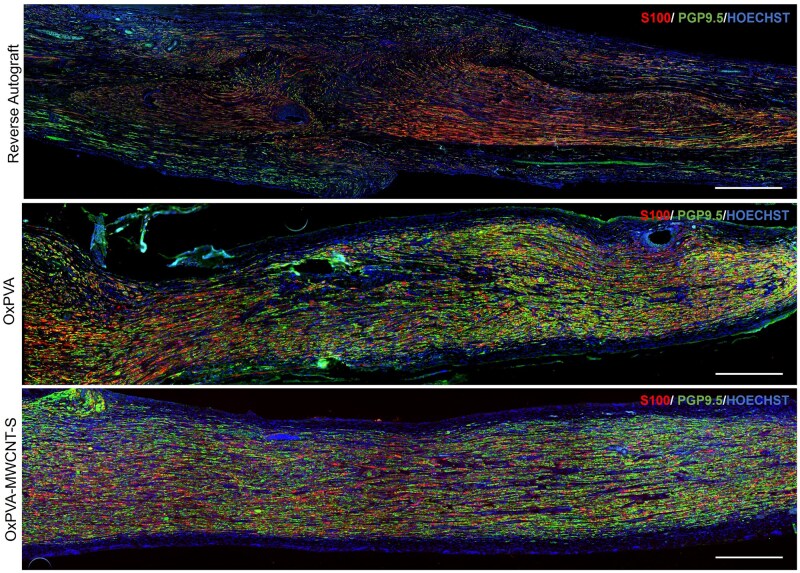
Immunofluorescence analysis of nerve regeneration after 6 weeks from surgery. Representative images of full‐length longitudinal sections of the explanted grafts/conduits displaying newly regenerated axons. Double immunofluorescent staining shows Schwann cells (S100, red) and axons (PGP9.5, green). Cell nuclei are stained with Hochest (blue). Scale bar: 500 μm.

Nerve regeneration through the synthetic devices was furtherly evidenced by longitudinal sections immunostained with S100 (red) and the peripheral nerve fibers marker PGP9.5 (green); the conduit, still well recognizable, supported the structural reconnection between the two stumps (Figure 10).

### Morphometric analysis

**Figure 11.**
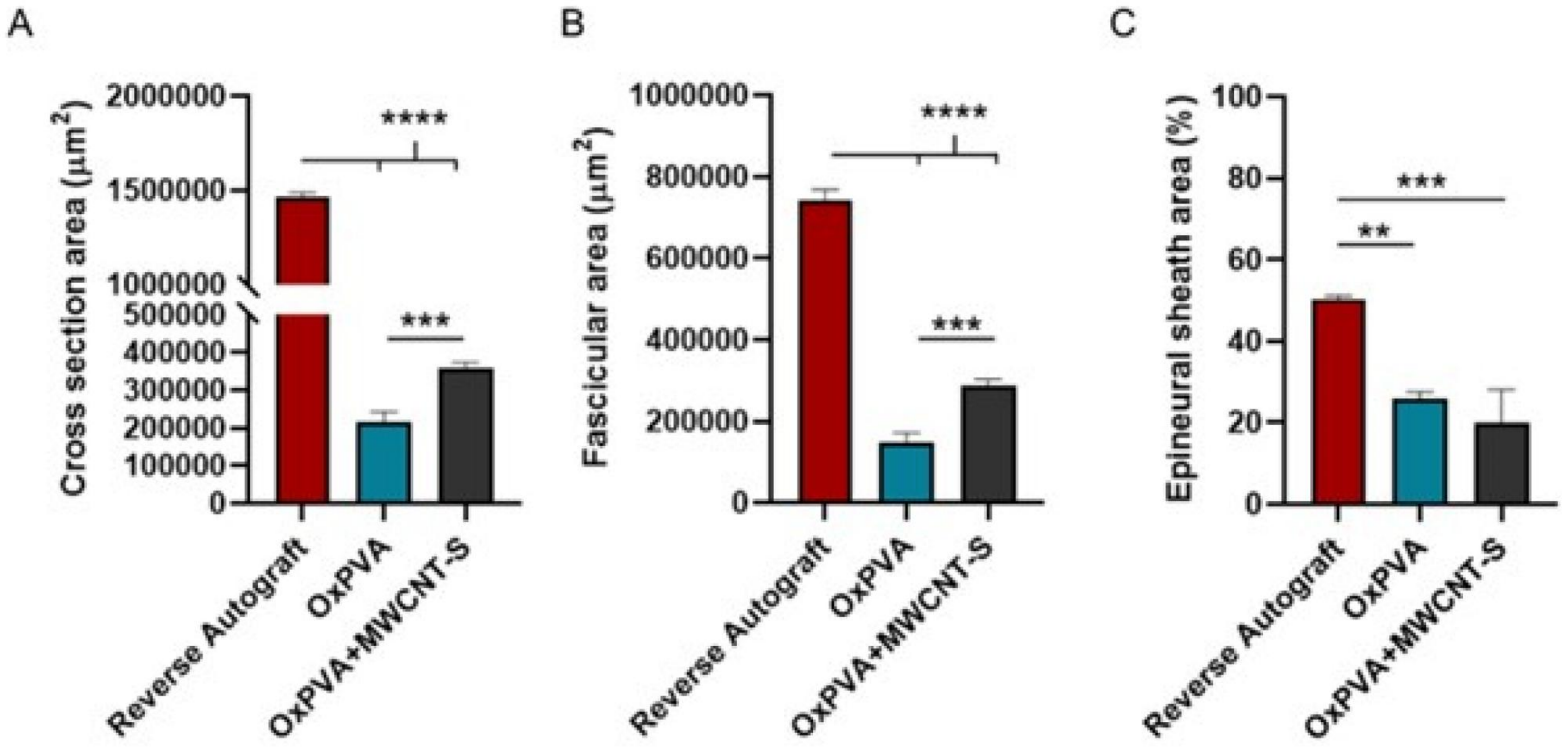
Morphometric analysis of Central transversal section. Histograms showing mean total cross-section nerve area (μm^2^) (**A**), fascicular area (μm^2^) (**B**) and epineural sheath area (%) (**C**). (**P < 0.01; ***P < 0.001; ****P < 0.0001).

Morphometric analysis is mandatory to compare repair strategies outcomes. Regarding the total cross-section area (μm^2^), that of Reverse Autograft (1 464 490 ± 22 771.18) was the highest (*P* *<* 0.0001) among that measured for all the other experimental groups (OxPVA: 216 776.1 ± 25 593.49; OxPVA+MWCNT-S: 359 149.1 ± 14 831.18) (OxPVA vs OxPVA+MWCNT-S: *P* < 0.001) (Figure 11A).

Similarly, focusing on fascicular area (μm^2^), Reverse Autograft group again showed the highest (*P* *<* 0.0001) mean area value within the whole cohort (740 261.7 ± 27 650.72) followed by OxPVA+MWCNT-S (286 320.8 ± 16 690.29) and OxPVA (147 745 ± 24 352.76). A statistically significant difference was also detected comparing OxPVA+MWCNT-S vs OxPVA (***P < 0.001) (Figure 11B).

Regarding the percentage (%) of epineural sheath thickness, the Reverse Autograft showed the highest values (50.42 ± 0.83) followed by OxPVA (25.91 ± 1.75) and OxPVA+MWCNT-S (20.10 ± 8.04). Significant differences were highlighted between Reverse Autograft and both OxPVA (*P* < 0.01) and OxPVA+MWCNT-S, respectively (*P* < 0.001) (Figure 11C).

**Figure 12.**
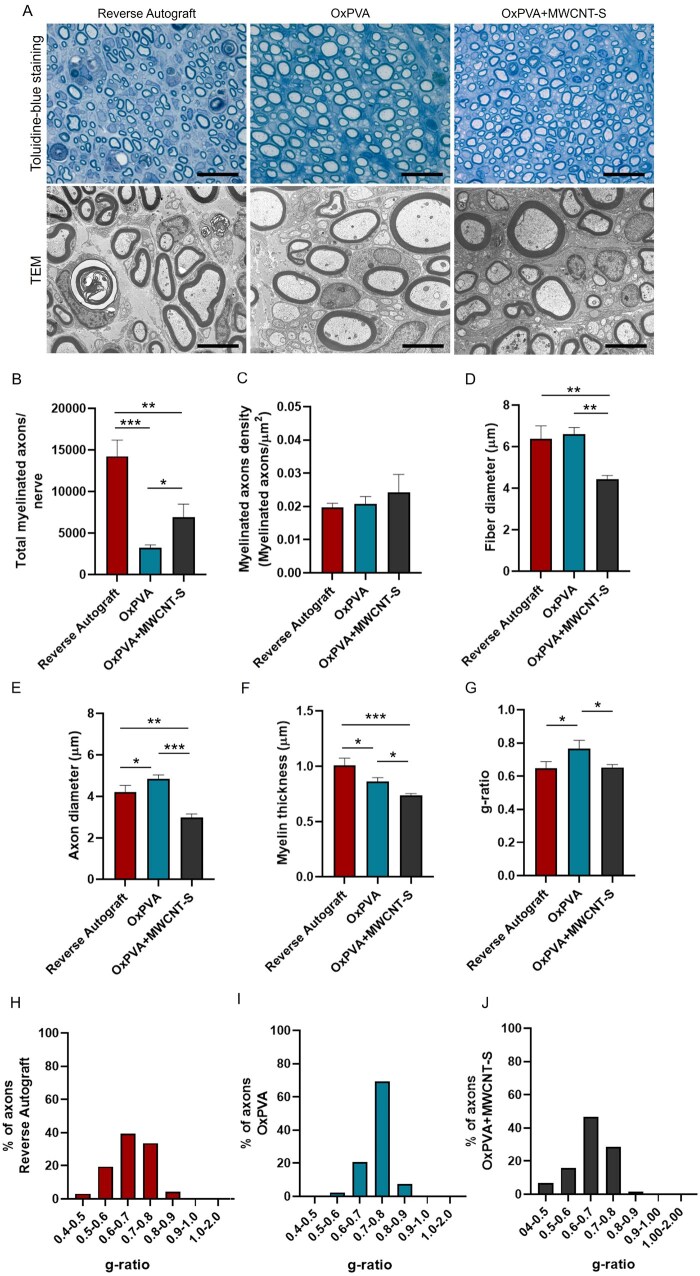
Myelinated axons analysis, Middle portion. (**A**) Regenerated tissue evaluation by toluidine blue staining and transmission electron microscopy (TEM) (scale bar: 20 μm (semithin sections); 6 μm, TEM images). (**B, C**) Histograms show total myelinated axons number/nerve (**B**) and myelinated axons density (number of axons/fascicular area (μm^2^)) (**C**). (**D-F**) Histograms show mean fiber diameter (μm^2^) (**D**), mean axon diameter (μm^2^) (**E**), mean myelin thickness (μm^2^) (**F**). (**G**) Mean g-ratio values calculated for each experimental group. (**H-J**) Assessment of g-ratio values distribution and percentage of prevalence for each experimental group. Results are reported as mean values ± SD (*P *<* 0.05; **P < 0.01; ***P *<* 0.001; ****P *<* 0.0001).

The myelination and axons regeneration were also focused by toluidine-blue staining and TEM (Figure 12A). Hence, total myelinated axons/nerve and myelinated axons density (myelinated axons/μm^2^) were determined. Thus, fiber diameter, axon diameter, myelin thickness and g-ratio were determined not only to provide a regenerated tissue description but also to give important data related to functional recovery.

Considering total myelinated axons number/nerve (nerve fascicle), Reverse Autograft showed the highest mean value (14 236.52 ± 1940.13) within the cohort (OxPVA, 3249.30 ± 349.89, *P* < 0.001; OxPVA+MWCNT-S, 6928.47 ± 1560.81, *P* < 0.01). A significant difference was also highlighted between OxPVA+MWCNT-S and OxPVA (*P* < 0.05) (Figure 12B).

As regards the myelinated axons density (axons/μm^2^) (Figure 12C), the morphometric study displayed this descending order: Reverse Autograft (0.019745 ± 0.001188)˃OxPVA (0.020826 ± 0.002151)˃OxPVA+MWCNT-S (0.024198 ± 0.005451); no significant differences were detected within the cohort.

Fibers’ diameter (μm) observed this trend: OxPVA (6.61 ± 0.30)> Reverse Autograft (6.37 ± 0.62) > OxPVA+MWCNT-S (4.44 ± 0.17); the OxPVA+MWCNT-S group displayed significant lower values than the other two experimental groups (*P* < 0.01) (Figure 12D).

Mean axons’ diameter (μm) was calculated for each experimental group: OxPVA, 4.84 ± 0.20*>* Reverse Autograft, 4.21 ± 0.33*>* OxPVA+MWCNT-S, 3.00 ± 0.17. Significant differences were detected comparing OxPVA vs Reverse Autograft (*P* < 0.05); OxPVA vs OxPVA+MWCNT-S (*P* < 0.01); Reverse Autograft vs OxPVA+MWCNT-S (*P* < 0.001) (Figure 12E).

Myelin sheath was measured (μm) and a progressive reduction in thickness was recognized according to the following order: Reverse Autograft (1.01 ± 0.07) > OxPVA (0.86 ± 0.04) > OxPVA+MWCNT-S (0.74 ± 0.02). The statistical analysis showed these significant differences: Reverse Autograft vs OxPVA and OxPVA vs OxPVA+MWCNT-S (*P* < 0.05); Reverse Autograft vs OxPVA+MWCNT-S (*P* < 0.001) (Figure 12F).

Additionally, the mean g-ratio (axon diameter/fiber diameter) was focused (Reverse Autograft, 0.65 ± 0.041; OxPVA, 0.76 ± 0.05; OxPVA+MWCNT-S 0.65 ± 0.02), significant differences were detected between OxPVA and the two other experimental groups (*P* < 0.05) (Figure 12G). As regards the g-ratio ranges distribution/prevalence (%) the interval 0.6–0.7 was mainly represented in the Reverse Autograft and OxPVA-MWCNT-S groups (39.47% and 46.81%, respectively) than in the OxPVA group (20.63%) which, in turn, mainly displayed the interval 0.7–0.8 (69.31%) compared to Reverse Autograft (33.68%) and OxPVA (28.72%) (Figure 12H–J).

### Gastrocnemius muscle weight

Gastrocnemius muscle atrophy, associated with a reduction in muscle weight, is commonly encountered following sciatic nerve transection. The data collected, comparing the percentage of wet weight of the gastrocnemius muscles in operated limb vs contralateral side, showed no significant differences between the experimental groups. The percentage values showed this descending order: Reverse Autograft (32.67 ± 23.70%) >OxPVA (30.33 ± 18.61%) > OxPVA + MWCNT-S (27.33 ± 4.93%).

## Discussion

Following peripheral nerve injury, the recovery of nerve function depends on the growth and extension of regenerated axons through the injured site until they reach and reinnervate the distal target organ [29]. Experimental evidence highlights conduit positioning as a promising alternative to autografts interposition. Tubular devices play a crucial role in guiding axons growth and providing a suitable microenvironment for release/diffusion of neurotrophic factors while also preventing fibro-connective tissue infiltration that can obstacle regeneration of a native-like tissue [30–32]. Within this context, emerging studies highlight the potential of conductive nanomaterials in peripheral nerve regeneration as they simulate the electrophysiological microenvironment, thus promoting neurons growth, differentiation and spatial organization [33–36]. Notably, nanomaterials incorporation within hydrogels, leading to ‘Hydrogel Hybrid’ [37], is an attractive approach to tailor hydrogels for specific end-use destination [38]. Despite several research groups have suggested the use of CNTs in peripheral nerve regeneration, their potential role within nerve conduits needs to be fully defined [39].

Hydrogels are physically/chemically cross-linked natural or synthetic 3D networks displaying properties that are close to those of living tissues, despite being hardly hydrosoluble [40]. They can be cast into various shapes and retain high amounts of water; this latter characteristic is responsible for their own elastic and soft consistency, together with a low interfacial tension. Interestingly, carbon-based nanomaterials (SWCNT/MWCNT and graphene) can provide conventional hydrogels with improved mechanical and electrical properties [38]. However, their strong agglomerating tendency together with intrinsic insolubility in common solvents represent the two main critical issues associated with them [41].

In previous works [25, 42] we have broadly described an efficient functionalization method (i.e. introduction of benzenesulfonate functional groups by diazotization reaction) that allowed MWCNT solubilization in water and dispersion in the synthetic polymer OxPVA. Mechanical incorporation of 0.1 wt% MWCNT-S within OxPVA solution led to cross-linked hydrogels showing shift in coloration (dark), increased surface conductivity (OxPVA+ MWCNT-S: 1.81×10^−6^±2.00×10^−7^ S; OxPVA: 2.00×10^−10^±9.16×10^−12^ S) and a more complex nanoscale topography by SEM [25]. However, effectiveness of OxPVA+MWCNT-S tubular devices whether implanted in animal model of peripheral nerve lesion was not considered, thus representing the purpose of this work.

Regeneration of the peripheral nerve following substance loss is a complex event which requires the careful orchestration of several factors and cues to establish the ideal microenvironment [43]. Current advances in development of nerve conduits aim to create an internal milieu mimicking the natural one to guarantee for a better therapeutic efficacy [44, 45]. Once physically cross-linked, OxPVA+MWCNT-S conduits confirmed a certain nanoporosity in section with also surface alteration; this ultrastructural organization may have an interesting role in promoting adsorption of ECM proteins that are released during regeneration [43, 46] including plasma exudate rich in neurotrophic factors and ECM precursor molecules (fibrinogen and factor XIII) [3]. As discussed by Ravanbakhsh *et al.* [47], hydrogel pore size changes when nanoparticles are introduced in the network; in case of OxPVA, this event may be ascribed to both MWCNT-S mechanical incorporation method and their interaction with OxPVA chains. Additionally, it can be assumed that increase in nanoporosity furtherly improves OxPVA ability in adsorb and release bioactive molecules [15, 20, 17] transforming the conduit luminal space into a regeneration camera for axons.

As mentioned above, hydrogels are considered highly suitable for nerve conduits fabrication due to their tissue-like main features (e.g. high water content, compliance porosity). However, their use is hindered because of scant mechanical behavior [48] affecting devices performances in guiding and supporting axonal growth [49]. According to previous evidence, this is not the case of OxPVA-derived conduits/wraps, showing to fit well the nerve end-stumps and resist at suture without giving rise to ruptures [2, 18, 19]. One of the mechanical requirements of a nerve conduit is to have an adequate SRS, which allows maintaining a structural continuity between the conduit and the stump during the regeneration process. The SRS of OxPVA and OxPVA+MWCNT-S varied between 0.23 and 0.27 N and between 0.12 and 0.23 N, respectively. These values are comparable with SRS values reported for other nerve conduits in the literature. For example, a double network hydrogel conduit based on gelatine and alginate developed by Kim *et al.* [48] showed SRS values varying approximately between 0.07 and 0.13 N, depending on the cross-linking method. In this work, both the wall thickness of the neural conduit and the suture thread caliber were close to the ones adopted in the present study. Moreover, our results highlighted that the incorporation of MWCNT-S into OxPVA hydrogel did not significantly modify SRS. This result confirmed that the mechanical tensile properties of the composite OxPVA+MWCNT-S at a concentration of 0.1% of carbon nanotubes are comparable to the mechanical properties of OxPVA alone [25].

After surgery, animals’ wellbeing was monitored along time. There is consensus in defining a body-weight reduction of 20% or more as severe suffering and thereby as a potential parameter for humane endpoint decisions [50]. Here, implants were well tolerated by whole cohort furtherly confirming previous evidence gathered for OxPVA [2, 18, 19] and suggesting the same also for the composite OxPVA+MWCNTs device.

Specific functional outcomes were assessed by SFI evaluation. Certainly, functional evaluations are difficult to carry on in animals but, despite some issues, the SFI method can be considered the most versatile approach (low cost, easy to apply) compared to other methods [51]. Generally, the normal SFI value is approximately 0 [26, 52], that is far from the results collected by the three study groups. However, statistical analysis confirmed no significant differences within the cohort, suggesting that OxPVA-based conduits provide functional outcomes comparable to those of the gold standard RA. It can be assumed that the low values recorded depend on the early time of evaluation: measurable functional improvements may be detected after longer follow-up periods. In support of this, the SFI data observed in this study are consistent with those previously reported in the literature for the same injury model [53–55]. Contextually, our histological and morphological data provide early indicators of regeneration, which may precede identifiable functional recovery.

Following functional recovery assessment, the sciatic nerves were observed *in situ* prior to retrieval. As reported in previous studies by Stocco *et al.* [2, 15, 18, 19], the OxPVA-based hydrogels remained identifiable at the implant site suggesting the devices adequacy in supporting regeneration. Peripheral nerve regeneration typically occurs at a rate of approximately 1–3 mm/day, depending on the location of the injury (proximal vs distal) [56]. The reaction stoichiometry employed to obtain 1% OxPVA was specifically developed to enhance the biodegradability of cross-linked PVA, which is otherwise poorly degradable after cross-linking. In fact, as reported by Gao *et al.* [57], an ideal nerve conduit should remain stable in structure until the gap is bridged, and distal stump is re-innervated. Following this, the device can gradually degrade. Within this context, there is consensus on the effects associated with improper degradation rates: whether too rapid it may result in swelling/inflammation; whether too slow, it can result in nerve compression, with eventual chronic immune rejection [57]. The degradation profile of 1% OxPVA aligns with these requirements, supporting its suitability for use in nerve conduits.

The regenerated tissue was evaluated through specific immunostaining/immunofluorescence analyses and morpho-structural evaluations. Immunopositivity toward markers for Schwann cells, axons and neurofilaments confirmed presence of nervous tissue inside the conduits; longitudinal sections (S100/PGP9.5 double labelled) corroborated the conjunction of the end-stumps. Additionally, epineurial tissue formation was highlighted by Azan Mallory (transversal sections, central portion); epineurium guarantees adequate microenvironment conditions that supports Schwann cells attachment and, therefore, promotes growth of axons with also angiogenic properties Siemionow *et al.* [58], 2020 as here confirmed by CD31 immunopositive elements. Angiogenesis is particularly important during reinnervation as it initiates nerve regeneration [59]. Intriguingly, blood vessels not only supply the tissue with blood, oxygen and nutrients, exerting a key role in peripheral nerve function but also provide a track for Schwann cell migration; this event is necessary for axonal pathfinding across the nerve bridge [60].

Materials based on CNT have been investigated for several biomedical applications. However, experimental data regarding CNT biocompatibility and toxicity in different biological environments are controversial: there are several studies showing that MWCNT-containing systems are innocuous, whereas others highlight their cytotoxicity. Together with CNT concentration, surface area, shape and size [61], their solubility in aqueous solvents is a prerequisite for biocompatibility thus avoiding their assemble in bundles due to strong hydrophobic and π–π interactions between tubes [36]. Following OxPVA+MWCNT-S conduits implantation, no severe inflammation signs were detected both macroscopically and after processing tissues (CD3; F4/80). Results were comparable within the cohort suggesting that the devices were all well tolerated, in accordance with previous evidence [25].

Morphometric study was finally performed focusing on cross-section area, fascicular area and epineural sheath area together with myelinated axons number, density, mean diameter, myelin sheath thickness and g-ratio [2]. Nerve regeneration within a hollow conduit starts with the formation of an acellular fibrin cable between the two stumps, allowing for cells (Schwann cells, endothelial cells, fibroblasts) migration along it. After cells migration, the fibrin cable undergoes degradation with onset of remyelination by mature ‘myelinating’ phenotype Schwann cells that wrap around the regenerated axons to form the myelin sheath [3, 62]. Fiber and axon diameter and myelin thickness are fundamental to study the regeneration outcome as affecting the conduction velocity of the nerve impulse. In particular, conduction velocity seems to be more related to axon diameter than total fiber diameter [63]; increasing axon diameters accelerate action potential conduction along the axons [64]. Regarding g-ratio (ratio of inner axonal diameter to the total outer diameter), the value 0.6 is of interest being associated with optimize nerve impulses conduction. Contextually, if g-ratio values are below 0.4, it may advise for degenerated nerve fibers characterized by abnormal myelin sheath thickening, whereas values higher than 0.7 may indicate either regenerated fibers with thinner myelin sheath or demyelinated nerve fibers [2, 62, 65]. Focusing on study evidence, higher values of mean axon diameter were observed for OxPVA vs OxPVA+MWCNT-S; the same was for fiber diameter and myelin thickness. However, despite this may suggest better functional outcomes with OxPVA NCs than composite device, it must be noticed that both the total myelinated axons/nerve and axons’ density are higher for the OxPVA+MWCNT-S group than OxPVA, possibly mitigating functionality assessment based on the sole g-ratio. Furthermore, g-ratio distribution highlighted for OxPVA+MWCNT-S and Reverse Autograft the same trend; most nerve fibers fell within the 0.6–0.7 interval (46.81% and 39.47%, respectively) followed by 0.7–0.8 interval (28.72% and 33.68%, respectively); differently, OxPVA distinguished for a 0.7–0.8 interval (69.31%) prevalence.

Certainly, including electrophysiological evaluation, outcomes referring to a longer period of *in vivo* implant, and data on a sham control group (here not provided and possibly representing a study limit), would be fundamental to fully assessing motor recovery in this sciatic nerve defect model. The lack of a sham group prevents direct comparison with native, uninjured nerve tissue; however, the inclusion of the Reverse Autograft group, widely regarded as a clinical gold standard, gives a benchmark for evaluating the regenerative performance of the OxPVA and OxPVA+MWCNT-S conduits against a standard approach broadly used in clinical practice. Despite these limitations, the experimental evidence gathered here represents a significant step toward the identification of more effective NCs for peripheral nerve regeneration, based on enhanced hydrogels incorporated with electroconductive nanomaterials.

## Conclusions

In this work, we explored and confirmed the potential of a new hybrid nanocomposite material for NCs fabrication, derived from the combination of OxPVA hydrogel with MWCNT-S. Interestingly, the incorporation of MWCNT-S (0.1 wt%) within OxPVA polymer solution proved to be effective: it was low-dose, homogeneous and did not affect/compromise the mechanical strength of the cross-linked material. Moreover, preclinical study evidence including histological, immunohistochemical and immunofluorescence analyses of the explants showed that the composite NCs promoted tissue regeneration with Schwann cells, axons and blood vessels that were all recognized, without signs of inflammation. These results are in accordance with previous evidence [25] where the presence of MWCNT-S into OxPVA was not associated with cytotoxicity *in vitro* or local signs of inflammation after subcutaneous implantation (14 and 42 days), proving composite material biocompatibility.

Regarding regenerated nerve morphometric analysis, lower values in terms of cross-section area and fascicular area were observed in OxPVA+MWCNT-S conduits compared to the Reverse Autograft group, though not in comparison with OxPVA alone. However, it should be noted that Reverse Autograft implantation does not represent a *de novo* regeneration process, as occurs with hollow conduits. This may explain the observed results, including the highest total number of myelinated axons per nerve in the Reverse Autograft group. The epineural sheath area (%) was most represented in the Reverse Autograft group, followed by OxPVA and OxPVA+MWCNT-S. The thickness of the epineurium in a regenerated nerve likely reflects the interplay between axonal regeneration, inflammatory activity and ECM remodeling. It may appear increased as consequence of fibrosis or protective mechanisms or reduced because of a diminished inflammatory or fibrotic response with a limited connective tissue deposition [66, 67]. Myelinated axon density was highest in the OxPVA+MWCNT-S group. Regarding morphometric axonal data used for g-ratio calculation, the g-ratio distribution showed a similar trend between the OxPVA+MWCNT-S and Reverse Autograft groups.

In the future, attention will be directed toward evaluating the performance of composite NCs in bridging larger nerve gaps (≥1 cm), as well as assessing their long-term efficacy and safety through extended follow-up studies to comprehensively evaluate functional outcomes. This approach will help to better understand their full therapeutic potential in clinically relevant scenarios. Moreover, incorporating electrophysiological assessments could provide a more accurate characterization of functional recovery over time across the cohort, in relation to the specific treatment.

## Data Availability

The datasets used and/or analyzed during the current study are available from the corresponding author on reasonable request.
